# Why Regulators Lost Track and Control of Pesticide Risks: Lessons From the Case of Glyphosate-Based Herbicides and Genetically Engineered-Crop Technology

**DOI:** 10.1007/s40572-018-0207-y

**Published:** 2018-07-12

**Authors:** Charles M. Benbrook

**Affiliations:** Visiting Scholar, Center for a Livable Future, Johns Hopkins University, 90063 Troy Road, Enterprise, Oregon, 97828 USA

**Keywords:** Glyphosate, Roundup, GMO, Pesticide regulation, Resistance, Pest management systems

## Abstract

**Purpose of Review:**

The approval of genetically engineered (GE) crops in the late 1990s triggered dramatic changes in corn, soybean, and cotton pest management systems, as well as complex, novel regulatory challenges. Lessons learned are reviewed and solutions described.

**Recent Findings:**

Government-imposed resistance management provisions can work and adapt to changing circumstances, but within the private sector, pressures to gain and hold market share have thus far trumped the widely recognized need for resistance management. Risks arising from the use of formulated pesticides often exceed by a wide margin those in regulatory risk assessments based on data derived from studies on nearly 100% pure active ingredients.

**Summary:**

Innovative policy changes are needed in four problem areas: excessive faith in the accuracy of pre-market risk assessments and regulatory thresholds; post-approval monitoring of actual impacts; risk arising from formulated pesticides, rather than just pure active ingredient; challenges inherent in assessing and mitigating the combined impacts of all GE traits and associated pesticides on agroecosystems, as opposed to each trait or pesticide alone; and, tools to deal with failing pest management systems.

## Introduction

Significant pest management system changes in the United States (U.S.) began in the mid-1990s, driven by the introduction of genetically engineered (GE) crops. Two commercially significant GE technologies have dominated the land area planted to GE crops over the last 20 years [1]: (1) the biosynthesis within GE plants and in vivo delivery of *Bacillus thuringiensis* (*Bt*) toxins in GE corn and cotton (so-called *Bt*-transgenic crops), and (2) transgenic crops able to survive post-emergent applications of the broad-spectrum herbicide glyphosate, aka Roundup, in the process controlling all, or nearly all, weeds growing in a field, while leaving agronomic crops largely unharmed.

There are now multiple herbicide-resistant (HR) traits in GE corn, soybeans, and cotton, and in recent years, corn and cotton have been engineered to express multiple *Bt* toxins, usually in conjunction with one or two HR traits, via what is called a “stacked” variety of corn or cotton. HR varieties of canola, sugar beets, and alfalfa are also now widely planted in the U.S. and some other countries [1].

For the first 5 to 10 years of use (1996–2005), these GE-seed-based technologies provided farmers new pest management options that were extremely effective, easy to deploy, robust, and roughly the same cost as alternative methods of dealing with the same weed and insect pests [2]. As a result, adoption was swift and near universal, often reaching or exceeding 90% of crop acres planted in the U.S. within the first decade after initial launch.

### Round One—a Solid Success and One Massively Consequential Miss

Pest management technologies come and go. In the last half-century, the major factor driving pesticides off the market has been the emergence and spread of pest phenotypes that have become resistant to once-effective pesticides. In most cases, slipping efficacy driven by resistance leads to a decision by pesticide manufacturers to phase out and replace a product that is not performing well, with a newly registered pesticide with equal or better efficacy. Problems arising from the spread of resistant pests also typically predate adverse regulatory actions by the Environmental Protection Agency [3].

In the early days of GE crop technology (1990–2000), the threat of resistance and what to do to prevent it received more public and regulatory attention than any other issue [4]. Key, initial regulatory actions included (1) the setting of pesticide residue tolerances [5••]; (2) approval of new pesticide-product labels authorizing post-emergent sprays on GE crop cultivars, often at higher rates and over more applications in season; and (3) approval (in USDA-terminology, “de-regulation”) of the seeds expressing new GE-transferred traits.

#### The Success—Mandatory *Bt*-Resistance Management and Annual Monitoring

Policy review processes in the first half of the 1990s focused on GE crop-related resistance management issues, and secondarily, gene flow from transgenic varieties to either sexually compatible wild relatives or other crops. In the case of transgenic, *Bt*-producing corn and cotton varieties, the EPA reached an agreement with the pesticide-seed-biotech industry, after years of dialog, on mandatory *Bt*-resistance management strategies. The USDA and grower groups went along with agreed-upon requirements with varying degrees of enthusiasm. Environmental and consumer groups generally applauded the unprecedented, pre-emptive effort to not just manage resistance, but prevent it all together [3].

The two most important requirements were adherence to the “high-dose strategy,” whereby, at least initially, the technology companies were required to show that their new *Bt*-varieties expressed enough *Bt* toxin in plant tissues to reliably kill 99.9% of the target insects [6]. Second is the refugia strategy, whereby farmers could plant only around three quarters of the acres in a given field to a *Bt*-producing cultivar, to assure survival of an ample number of still-*Bt*-susceptible insects on the other, ~one quarter of each field [6].

The hope and expectation was that the high-dose plus refugia strategy would assure an ample supply of still-susceptible insects in crop fields, and that these insects would mate with the few insects surviving to adulthood in the portions of fields planted to GE-*Bt* seeds [6]. The end result would be a genetic dead end, in cases where a mutation allowed a few insects to survive intakes of *Bt* toxin lethal to nearly all other insects.

Importantly and in addition, the EPA imposed on the industry and growers well-crafted, mandatory, and funded insect-resistance monitoring requirements [6]. Post-approval surveillance was justified by the recognition that *Bt*-resistance management “plans” were experimental and would likely require mid-course corrections [3, 6].

The plans worked well, indeed perhaps too well, since industry and grower pressure to relax the refugia requirement began building in the mid-2000s. The EPA was forced politically to incrementally relax, or no longer require the two core pillars of existing *Bt*-resistance management plans because, supposedly, they were no longer needed [7]. But as many independent entomologists realized and argued at the time, the strategies were just as vital in the mid-2000s as they were in 1996, and the risk of resistance would rise in step with the relaxation of the successful strategies that had been developed to contain it [6, 8•, 9].

Routine, mandatory monitoring soon proved they were right. The emergence and spread of resistant cotton and corn insects have markedly reduced the value of the *Bt*-transgenic traits, and, in the absence of changes in policy and practice, will eventually render them obsolete, including those expressing multiple *Bt* toxins [9].

This regrettable outcome will impose large costs on farmers and society. The collapse of *Bt*-transgenic technology was almost assuredly avoidable had the industry and EPA re-imposed previously effective strategies largely abandoned a decade ago. It still might be avoidable, if appropriate, resistance management-driven changes are made, and soon, in the way *Bt*-transgenic crops are now used. Unfortunately, such an outcome is unlikely given the current political climate in the U.S.

The absence of laws, regulations, and policies sufficient to avoid the collapse of *Bt*-transgenic technology is now recognized [8•, 10, 11]. Many concrete proposals have been advanced by the National Academy of Sciences [6–8] and other groups [3, 9, 11] in the hope of turning the tide on resistance. But over most of the last decade, there has been little serious discussion in Congress or federal agencies on how to build pesticide- or GE trait resistance management into the mandatory provisions embedded in pesticide labels and GE-seed technology use agreements.

#### A Big Miss—EPA’s Failure to Require Mandatory Glyphosate-Resistance Management Practices and Systematic Resistant-Weed Monitoring

In the first half of the 1990s, another GE crop policy dialog unfolded, this one focused on preventing resistance to herbicides linked to GE-HR crops, and especially Roundup Ready (RR) crops [3, 7]. Many of the same stakeholders involved in the debate over *Bt*-resistance management also engaged in discussions over whether and how to prevent the emergence and spread of herbicide-resistance weeds, if and when emerging GE-HR-RR cultivars were approved and widely planted.

Monsanto, the manufacturer of Roundup, argued there was no cause for concern over resistance because of glyphosate’s 20+ years of use in the U.S., with no evidence of serious problems with resistance weeds [12–14]. As glyphosate-resistant weeds emerged and spread in the 2001–2015 period, Monsanto scientists and their allies in academia and the farm community then pointed out that most other widely used herbicides had triggered the spread of resistant weeds, so the emergence of glyphosate-resistant weeds was nothing new, nor different. But the underlying factors driving the emergence and spread of glyphosate-resistant weeds, and the economic and agronomic costs of failure to curtail their spread were different from any past herbicide-resistant weed challenge.

Herbicide-resistance technology allows multiple applications over extended periods of time, thereby markedly increasing selection pressure on weed populations. When farmers plant RR crops year after year (e.g., RR corn following RR soybeans in continuous rotation), weed populations are hit multiple times annually with just one active ingredient, maximizing the chance of a genetic mutation leading to the spread of resistant phenotypes [3, 8•]. Simplifying a complex phenomenon, on a scale of 1 to 10, in the pre-GE crop era, the risk of resistance occurring from major, pre-emergent uses of glyphosate was around 1, but given the way the herbicide would be used in GE cropping systems, the risk of resistance rose close to 10.

Few are aware that in public meetings convened by EPA in the early 1990s to discuss GE-HR-RR crops, prior to their approval, some environmental and public interest group representatives advanced an argument in support of mandatory, glyphosate-resistance management provisions for basically the same reason that they had supported similar, mandatory *Bt*-resistance management plans [3, 7].

The common reason was to preserve the efficacy of both GE technologies, in light of their inherent, positive attributes, and the hope that they would reduce reliance on almost assuredly more toxic pesticide alternatives. Both technologies did just that for a period of time, but the spread of resistant target pests has incrementally eroded the efficacy of both technologies. Today, considerably more herbicide is needed on fields planted to GE-HR crops than prior to the launch of RR technology, and farmers are advised to spray insecticides and plant insecticide-coated seeds to prevent the further spread of insects resistant to *Bt* [5, 7, 8•].

#### Why One Hit and a Miss?

There are various theories explaining why EPA accepted and acted upon the arguments advanced in support of mandatory resistance management in the case of *Bt*-transgenic crops, but not in the case of GE-HR-RR crops. The most plausible explanation is that *Bt*-susceptibility genes in target insect populations can be characterized as a gift from nature to mankind, and one for which no private company has a right to degrade.

Furthermore, as some people argued, if a company did trigger *Bt*-resistance in a major, widely dispersed insect pest, the company should be held responsible for the consequences, including future losses of fruit and vegetable crops for which *Bt*-foliar sprays were previously the foundation of worm control programs [3].

But glyphosate and formulated Roundup herbicides were different from *Bt*-transgenic crops. The technology was clearly not derived from nature. Glyphosate had been synthesized by a chemical company, recognized as an active herbicide, and then patented and developed by Monsanto. EPA took the position that pesticide manufacturers had good reasons to estimate the risks and costs of resistance, including lost sales [16], and were in the best position to research and develop effective, resistance management plans. The agency assumed that resistance management would maximize long-term sales and profits, and so expected manufacturers to impose label restrictions sufficient to prevent resistance as a routine part of “product stewardship.” Unfortunately, this assumption was not grounded in reality, as events in the field proved.

The first glyphosate-resistant weed was confirmed in a RR soybean field in 2001, 5 years after commercial launch of RR technology and roughly on schedule as predicted in the mid-1990s [3, 9]. By the end of 2005, scientists had confirmed the presence of glyphosate-resistant Palmer amaranth in several southern states. Since 2005, it has been clear that RR technology created a near-perfect storm to accelerate the emergence and spread of glyphosate-resistant weeds.

But even when the seriousness of the resistant threat facing RR technology was obvious to farmers, academic weed scientists, and the pesticide industry, EPA took no action. Another major player in the pesticide-biotech-seed industry, Syngenta, stepped forward and tried to broker an industry-wide, glyphosate-resistance management plan. To work, such a plan would have to be adhered to by all registrants of glyphosate-based herbicides, including Syngenta, the registrant of Touchdown herbicide in which glyphosate was the active ingredient.

The crux of the plan was simple. It would have required that glyphosate-based herbicides could not be applied on the same field in no more than 2 years out of any 3. Plus, it was implicit in the plan that if resistant weeds continued to spread, then the restriction on the frequency of glyphosate use would be tightened to once every 3 years, or 4 years, until annual monitoring showed no further spread of resistant weeds, or better yet, fewer glyphosate-resistant weeds overall.

If Syngenta’s plan had been adopted industry-wide and implemented in step with the scope of the problem, the RR-crop technology meltdown of the last decade could have been curtailed, if not avoided altogether. But back when it really mattered, Monsanto refused to back Syngenta's plan, and it quickly sank below the radar screen.

Since the Syngenta-led effort, there have been no meaningful efforts by the industry to address the underlying factors driving the emergence and spread of GE crop-driven herbicide-resistant weeds. The pesticide-seed-biotech industry’s solution to the problem, as reflected in their R+D investments over the last decade and new GE-HR crop introductions in the last few years, is second-generation GE-HR crops that are engineered to resist multiple herbicides [5, 8•, 18].

Moreover, most farmers will double-down on HR technology whether they want to or not, because the biotech-seed companies control the traits that they will move into the most popular crop genetics. In crop year 2018, around three quarters of the soybean seed offered to farmers will express the glyphosate-resistance gene, plus either dicamba or 2,4-D resistance genes. Some cultivars planted in 2018 will be resistance to five or more herbicides. Companies have patents on GE-transformation systems conferring resistance to 10 or more herbicides.

As a result, there will be more herbicide use, triggering the emergence and spread or new resistant weeds that will, in turn, require more applications of now several herbicides [5••, 15•, 18]. The rising number of herbicides applied, more applications, and generally higher rates of application will generate record sales and profits for companies selling premium-priced GE-HR seeds and traits, and the herbicides required to bring these crops to harvest.

So, the failure to prevent resistance in the first decade of GE crop use (1996–2005) set the stage for the collapse in first-generation GE-HR crop technology, thrusting the herbicide treadmill into a higher gear.

The consequences are clear. Seed-plus-pesticide costs are taking a much larger share of per-acre crop income on most farms reliant on GE cultivars [5••]. Overall pesticide use has about doubled, led by the huge increase in the volume of glyphosate applied, coupled with the growing need for two to four or more additional herbicides to deal with infestations of glyphosate-resistant weeds [5••, 15•, 18, 19••].

Herbicide residues in food were rarely a concern in the past, because most herbicides were applied either before a crop had germinated or soon thereafter, and in any event, months before the harvested part of the crop had begun to form. But today, herbicide residues in food are a growing concern because of late-season applications on GE crops, and use of glyphosate as a pre-harvest desiccant applied to speed up harvest operations on wheat, other small grain, and certain other crop farms [5••].

A growing portion of water resources in heavily farmed areas in the U.S. has one to six herbicides and/or herbicide metabolites in it for a good portion of the year, if not year round. The USDA’s Pesticide Data Program tested groundwater for several years in the late 2000s. In their 2009 program year annual summary report, they report results of 278 groundwater samples. Among the private residence wells tested, five herbicides were found in 40% or more of the wells tested. Three were present in 70% or more. Four metabolites of atrazine were found in 58.6, 51.4, 27, and 58.6%, and parent atrazine was reported in 45.7% [20]. Glyphosate is now found in most samples of surface, and many groundwater samples throughout the Midwest [21•].

As a result, risks of long-term chronic diseases, reproductive problems and birth defects, and heritable genetic changes among women and infants living in heavily farmed regions are rising [22••, 23••]. Yet most farmers see no viable alternative for dealing with today’s weed management challenges, given the scale of their farming operations and the resources they have at hand.

### Key Gaps in Regulatory Law, Policy, and Risk Assessment Tools

In just the last few years, there has been a surprising convergence of views on the most important problems arising from corn, soybean, and cotton pest management systems and pesticide use. Important lessons have been learned from the rapid adoption, slipping efficacy, and rising costs and risks associated with GE-HR and *Bt*-transgenic technology [8•, 11, 15•, 17, 18].

The sharply upward trajectory in herbicide drift and damage arising in the wake of growing use of dicamba and 2,4-D in conjunction with Roundup is triggering new concerns and tensions. Herbicide drift and movement leading to non-target crop damage adversely impacted around 5 million acres in 2017 and pitted farmers against neighbors in hundreds of communities (for state-by-state details, see “Dicamba Watch” [24]).

Events unfolding as a result of the planting of dicamba-resistant soybeans and cotton in 2016 and 2017 in Arkansas, Missouri, and Tennessee, and to a lesser extent in nearby states, are a harbinger of problems likely to unfold in the heart of the Cornbelt in the next few to 10 years. Moreover, the roots of the problems experienced in 2016–2017 are grounded in gaps now evident in the laws, regulations, and tools available to federal and state agencies responsible for keeping up with rapidly evolving weed management system challenges and herbicide use and risk trajectories.

Another lesson has emerged. Some of today’s laws and policies, and priorities are pushing farmers in the wrong direction. Others virtually guarantee that regulators will continuously be behind the curve in recognizing, much less addressing or preventing, major increases in risks and collateral damage.

Today’s problems are rooted in poorly conceived policies that are incapable of response in real time to keep small, emerging problems from becoming big, long-lasting ones. In addition, it is vital to stress that risk mitigation after the fact is a sign of policy and technology failure, not success.

Until there are significant changes in law and policy and/or private sector priorities, today’s systemic failures will persist, unless farmers decide to move on their own away from near-sole reliance on herbicides by adopting multi-tactic, integrated weed management systems, as recommended by academic weed scientists and professional societies [15•, 18, 25].

#### Empowering Regulators to Meet Emerging Challenges

For regulators to gain the information needed to more accurately quantify and mitigate risks in real time, changes will be needed in law and policy. Some changes are simple and will be easy to implement (e.g., limiting use of glyphosate to no more than 2 out of 3 years on any given field), while others will require substantial reforms and investments in research (e.g., dealing with combined effects across all herbicides applied).

Unfortunately, the risk assessment tools, data, and models accessible to tackle important shortcomings in current regulatory policies and programs are sorely lacking [6, 8•]. But like when GE crop technology was first advanced for commercial use, regulators are not able to delay action while needed research can be undertaken. No action sometimes proves more costly than modest steps that are, at least, directionally correct, as the initial *Bt*-resistance monitoring and management plans proved to be.

A full list of generic shortcomings in agricultural biotechnology and pesticide regulation would be a long one, but five of the most important are described below. These arise in one of two major phases of GE crop and pesticide regulatory decision-making:

- Actions and decisions that occur before the first commercial use, including assessment of studies submitted to regulators, setting chronic and acute reference doses for pesticides, action on petitions seeking the establishment of tolerances covering unavoidable residues in food, and the conditions of use and safety precautions required on approved pesticide-product labels, including any specific actions needed to prevent resistance or spare harm to pollinators, and
- Re-assessment of risks and benefits, based on how GE traits and pesticides have actually been used, how frequently and widely they have been used, interactions with other chemicals (e.g., liquid fertilizers in tank mixes), whether and to what extent residues of chemicals or toxins make it into food as eaten, persist in soil, water, and the air, and/or impact non-target organisms or ecological cycles and interactions.

#### Five Challenges and Possible Solutions

##### Excessive Confidence in the Precision of Pre-approval Risk Assessment Methods

Experience suggests there is often a sizable mismatch between the risks of concern to regulators prior to a product’s first approved uses, and the risks that arise from the way a pesticide or GE trait is used once on the market. The impacts of neonicotinoid insecticides on pollinators and biodiversity are a good example [26•].

The limits of pre-market testing and risk assessment need to be acknowledged, and greater weight placed on post-approval monitoring that produces the hard data needed to refine risk assessments, detect resistant populations, and track levels in soil, water, and people [6, 7, 8•]. Such monitoring will lead to more solid measures of the presence of resistance genes in target-pest populations, environmental loadings, residues in food, dietary exposures, and chemical or toxin levels in human urine and blood. Monitoring also creates the ability to track trends, recognize inflection points, and determine the effectiveness of past risk-mitigation interventions.

##### The Current Focus in Risk Assessments on Pesticide Active Ingredients (e.g., Glyphosate or Imidacloprid), Instead of Formulated Pesticide Products (Roundup or Admire)

Perhaps the most obvious and consequential failure of pesticide-product testing and regulation is the near-sole focus on pure active ingredients, as opposed to formulated pesticide products [27, 28]. Clearly, Roundup herbicides are more toxic to many, if not most organisms, than pure glyphosate [28, 29]. In addition, formulated Roundup behaves much differently in the environment than pure glyphosate. Last, many of the so-called inert ingredients in formulated pesticides, including Roundup, are themselves toxic [30•], and/or enhance the ability of the active ingredient to persist in biological systems and penetrate cell walls.

Collectively, these are among the major reasons why the real-world environmental and public health impacts of pesticides, as applied, often differ so much from what regulators project, and strive to mitigate, when granting initial, *active-ingredient*-based approvals.

Similar active-ingredient versus formulated-product differences in risk profiles arise in the case of neonicotinoid (neonic) insecticides, the most widely applied family of insecticides in the world (e.g., imidacloprid, clothianidin, thiamethoxam). Another layer of complex risk assessment and regulatory challenges arise from the sometimes highly synergistic interactions of formulated neonic insecticides and several, widely used fungicides [31].

The lack of focus on formulated-product risk creates a blind spot around the risk profiles of the world’s most widely used herbicide (glyphosate) [5••], the most widely used insecticides (neonics), as well as many fruit and vegetable crops routinely treated at roughly the same time with synergistic fungicides and insecticides. This is why contemporary risk assessments based on pure active ingredients are so often unreliable.

This shortcoming has been recognized for decades, but meaningful reforms have been resisted globally, based on the argument that the cost, scope, and complexity of testing all formulated products would be untenable. The solution, however, is clear and, if implemented wisely, would not markedly increase the overall cost of pesticide regulation.

First, regulators should simply ban, or very heavily restrict, surfactants and adjuvants that are known to pose possibly substantial risks; the list of such inert ingredients is generally known, and not long.

Second, regulators should agree on a process to establish a global “Generally Recognized as Safe” (GRAS) directory of inert ingredients eligible for use in formulated, end-use pesticide products (i.e., farmer- and consumer-ready for use). Any formulated product containing only GRAS-listed inerts would be exempt from the requirement for additional testing, until and only if evidence emerges pointing to a need for such added testing.

There is a current, USDA-approved list of inert ingredients allowed in the formulation of biopesticides approved for use on organic farms [32]. This well-vetted list is a logical place to start in compiling the GRAS list of inert ingredients. Just as there is a defined process and set of testing requirements to register a new pesticide, a company wanting to get a new inert ingredient onto the GRAS list would have to fulfill applicable data requirements, and fit within the risk thresholds applicable to inert ingredients on the GRAS list.

End-use products manufactured with inert ingredients not on the GRAS list would have to go through a tiered-testing regimen, which in extreme cases might even entail 2-year, chronic feeding/oncogenicity studies in mice and/or rats. For example, the classification of glyphosate/Roundup herbicide as a probable human carcinogen in 2015 by the International Agency for Research on Cancer [33•], while the EPA continues to regard technical glyphosate as “not likely” to pose cancer risk [34•], highlights the importance of independent testing of both active ingredients and formulated products.

##### Lack of Post-approval Monitoring of Real-World Performance and Impacts

Post-approval monitoring should be thorough and rigorous, and mandatory for every new pesticide and GE trait. Initial focus should be on the most widely adopted food crop uses, and the regions where the highest percent of crop acres are treated or planted.

The timing and scope of such data collection should be a function of adoption thresholds, rather than in accord with an arbitrary number of years. For example, when a newly registered pesticide or GE crop trait is approved and comes to be used on, say 5% of national crop acres or 10% of the acreage in any given state, monitoring requirements should kick in.

The next two production seasons should be utilized to collect real-world data. Then, drawing on the new information generated, regulators should revisit and update their risk assessments, and determine whether any previously unforeseen risks warrant attention, and proceed accordingly.

In the case of pesticides or GE traits that have been on the market for several years, environmental, human exposure, and biomonitoring data should be collected in accord with a schedule driven by adoption thresholds and trends, coupled with observed, real-world impacts like levels in human urine or blood, epidemiology data, and number of resistant pest species, rather than arbitrary regulatory thresholds and time periods (e.g., the 15-year re-registration cycle in U.S. federal pesticide law).

Questions will arise over who should pay for such monitoring, who will conduct it and have access to the data, and what will happen when unexpected risks are recognized. But when new or heightened risk concerns arise, next steps should be decisive and occur in time to assure that risks and/or economic costs do not spiral out of control, as both appear to be doing in today’s GE-based weed management systems in several parts of the U.S.

When unexpected risks emerge, field-based monitoring data will give the industry, farmers, scientists, and regulators a jump-start on collecting the data needed to deploy targeted risk-mitigation measures. If such measures are agreed upon and implemented in a timely way, the need for more draconian interventions may never arise. This is clearly one valuable lesson learned from the different ways the threat of resistance was dealt with in the case of GE-*Bt*-transgenic crops, in contrast to GE-HR crops in the U.S.

##### Failure to Manage the Collective Impact of Pest Management Tactics, Pesticides, and Bt Toxins on Food Safety, Human Health, and the Environment

Whack-a-mole is an inefficient and usually futile regulatory approach. Reliance on insecticidal poisons in corn and cotton insect pest management systems progressed from the chlorinated hydrocarbon (OC) insecticides in the 1950s through early 1970s, and then to the organophosphate (OP) and carbamate insecticides in the 1970s and into the 1980s [3]. As OP and carbamate efficacy slipped because of the spread of resistance, the synthetic pyrethroids came along and gained market share. In the mid-1990s, resistance-driven, insecticide-control failures created strong demand for *Bt*-transgenic cultivars, technology which once again changed the nature, magnitude, and distribution of control costs, benefits, and risks.

But throughout the past half-century of insect pest management system change, farmers have become progressively more dependent on insecticide and toxin-based interventions, and incrementally more divorced from prevention-based, integrated systems that rely mostly on management of ecological interactions and biological control mechanisms.

From a technical perspective, it would be relatively straightforward to approximate the 5-year, rolling average number of herbicide, insecticide, or fungicide “kill units” required to bring a crop to harvest on a given type of farm in a given year, as well as over time.

For crops and in places where the average “kill unit” trajectory begins to slope upward, it should be clear to most everyone that a change in pest management tactics and strategy is now, or soon will be, in order. Likewise, for crops and regions where the “kill unit” curve is steady or sloping downward, all stakeholders in the system should be encouraged by the fact that existing pest management systems and technology seem to be working incrementally better, or at least as well as in the past. The need to alter pest management-driven risk trajectories, or sustain positive trends, leads to challenge number five.

##### Lack of Tools to Stabilize and/or Reverse Rising Risk Trajectories, Even When the Steps Needed to Do So Are Well Known

While the pest management knowledge and technology accessible to U.S. farmers is, in many respects, the envy of the world, there is remarkable variability in actual pest management system performance, costs, and associated risk profiles. The most dramatic differences arise when comparing the human health and environmental risks arising from pest management systems on conventional farms, in contrast to organic farms, where prevention is the foundation of management systems.

In the U.S., the EPA and USDA need new tools and authority to mitigate risks across all pesticides, tactics, and GE traits deployed in a given crop against a specific pest, or class of pests. Currently, these agencies can only focus on one pesticide or GE trait at a time and make their judgements based on whether a new technology appears acceptably safe, in isolation, on a given field. They do not consider the scope and scale of adoption. Once approved, a newly registered pesticide or GE trait can be used on 0 to 100% of crop acres, a range that has obvious implications for risk profiles and magnitude.

There are a variety of ways through which agencies could begin to reduce system-generated risks, as opposed to single pesticide or trait-induced risks: imposing similar pre-harvest intervals on all herbicides registered for uses known to lead to higher residues (e.g., pre-harvest, desiccation, or post-emergent sprays on GE-HR crops); comparable limits on the number of applications and/or rates; mandatory resistance management practices; and, comparable, reductions in tolerances to prohibit applications known to sometimes result in relatively high residues in food or animal feed.

## Conclusions

The goal of pest management-related research, farmer-support services, and regulatory programs and initiatives ought to be continuous improvement, measured by incremental progress in three metrics:

1. The efficacy of pest management systems
2. Pest management system costs
3. 5-year, rolling average number of “kill units” needed to bring a crop to harvest

Through the tracking of such metrics, pest management system performance can be monitored for changes and compared across systems, regardless of the type (conventional, GE-reliant, organic), location, or size of a farm. The efficacy of specific interventions can be tracked, sharpening understanding of what works and is affordable, versus efforts that deliver spotty or temporary benefits.

The rising costs and risks of toxin-based pest management, and tensions and controversies over pesticides and GE crops, will likely grow more acute. Market forces will likely continue to support investments in organic and other biologically based pest management systems, especially in countries with relatively high disposable income. Over the next half-century, farmer and consumer demand for more assuredly safe food and prevention-based pest management systems will eventually slow down the pesticide/toxin treadmill, but the costs to society in the interim may remain sizable.
